# Health care delivery system contributions to management of newly diagnosed prostate cancer

**DOI:** 10.1002/cam4.6349

**Published:** 2023-07-20

**Authors:** Noah Krampe, Samuel R. Kaufman, Mary K. Oerline, Dawson Hill, Megan E. V. Caram, Vahakn B. Shahinian, Brent K. Hollenbeck, Avinash Maganty

**Affiliations:** ^1^ Dow Division of Health Services Research, Department of Urology University of Michigan Ann Arbor Michigan USA; ^2^ Division of Hematology/Oncology, Department of Internal Medicine University of Michigan Ann Arbor Michigan USA; ^3^ VA Health Services Research & Development, Center for Clinical Management Research, VA Ann Arbor Healthcare System Ann Arbor Michigan USA; ^4^ Division of Nephrology, Department of Internal Medicine University of Michigan Ann Arbor Michigan USA

**Keywords:** delivery system, prostate cancer, variation in treatment

## Abstract

**Background:**

Despite clinical guidelines advocating for use of conservative management in specific clinical scenarios for men with prostate cancer, there continues to be tremendous variation in its uptake. This variation may be amplified among men with competing health risks, for whom treatment decisions are not straightforward. The degree to which characteristics of the health care delivery system explain this variation remains unclear.

**Methods:**

Using national Medicare data, men with newly diagnosed prostate cancer between 2014 and 2019 were identified. Hierarchical logistic regression models were used to assess the association between use of treatment and health care delivery system determinants operating at the practice level, which included measures of financial incentives (i.e., radiation vault ownership), practice organization (i.e., single specialty vs. multispecialty groups), and the health care market (i.e., competition). Variance was partitioned to estimate the relative influence of patient and practice characteristics on the variation in use of treatment within strata of noncancer mortality risk groups.

**Results:**

Among 62,507 men with newly diagnosed prostate cancer, the largest variation in the use of treatment between practices was observed for men with high and very high‐risk of noncancer mortality (range of practice‐level rates of treatment for high: 57%–71% and very high: 41%–61%). Addition of health care delivery system determinants measured at the practice level explained 13% and 15% of the variation in use of treatment among men with low and intermediate risk of noncancer mortality in 10 years, respectively. Conversely, these characteristics explained a larger share of the variation in use of treatment among men with high and very high‐risk of noncancer mortality (26% and 40%, respectively).

**Conclusions:**

Variation among urology practices in use of treatment was highest for men with high and very high‐risk noncancer mortality. Practice characteristics explained a large share of this variation.

## Graphical abstract

Despite increasing use of conservative management for men with prostate cancer, there is tremendous variation in its uptake. This variation may be amplified by features of the delivery system, particularly among men with competing health risks, for whom treatment decisions are challenging. In this study, we find that variation in use of treatment was highest for men with high and very high‐risk noncancer mortality. Characteristics of the delivery system, measured at the level of the practice explained a large share of this variation.

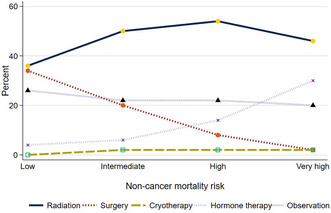

## 1 INTRODUCTION

Prostate cancer is the second most common malignancy among men, with more than 1.2 million cases reported annually, worldwide.^1^ Although clinical guidelines provide guidance,^2^ the decision to pursue treatment versus conservative management after a new diagnosis of prostate cancer can be challenging, particularly for men with significant competing health risks (i.e., high risk of noncancer mortality).^3^, ^4^, ^5^ Unlike young, healthy men, for whom treatment decisions are primarily dictated by tumor biology, assessment of competing risks must factor into the decision making for men with underlying health issues. The need to weigh the benefits of treatment against the risk of mortality due to competing risks—which is imprecisely estimated by physicians—adds significant medical uncertainty.^6^ Such decisions are discretionary and determined by both patient and physician preferences,^7^ and manifests clinically as variation in population‐based treatment rates.^8^ In these circumstances, variation may be further amplified by the influence of nonclinical factors.

In the most unhealthy population, health care delivery system determinants, such as those attributable to the physician or their practice, have the potential to influence behavior and subsequent decisions to treat.^9^ For example, urology practices with ownership interest in radiation vaults, whereby they collect both the professional and technical components of payment and thus have strong financial incentives, were more likely to use treatment, and radiation in particular, across all disease risk strata.^10^ Furthermore, physician organization (i.e., single specialty vs. multispecialty groups) is known to affect the uptake of conservative management, with small single specialty practices exhibiting the most rapid adoption.^11^ The influence of health care delivery system determinants, can open the door to overtreatment in circumstances in which treatment is most discretionary. Therefore, understanding the extent to which these health care delivery system determinants contribute to variation in use of treatment for prostate cancer is important for understanding the possible mechanisms through which policies may be designed to improve the quality of care.

For this reason, we performed a national study of Medicare beneficiaries with newly diagnosed prostate cancer to understand the extent to which health care delivery system determinants contribute to variation in use of treatment by noncancer mortality risk.

## 2 METHODS

### 2.1 Data and study population

We performed a retrospective cohort study of men with newly diagnosed prostate cancer using a 20% sample of national Medicare claims for fee‐for‐service beneficiaries between 2014 and 2019 with at least 1 year of follow‐up (i.e., through December 31, 2020). Men with newly diagnosed prostate cancer were included in the study using a previously established and validated method (specificity of 99.8%; positive predictive value of 88.7%).^12^ To limit ascertainment bias, men were excluded if they did not have continuous enrollment in Medicare Parts A or B the year before and after diagnosis or if they participated in Medicare Advantage. The cohort was further limited to men aged 66 years and older to allow for assessment of health status for the year preceding diagnosis. All men were then attributed to their primary urologist using methods that account for the both frequency and intensity of interaction surrounding the diagnosis.^10^, ^12^ Urologists were further attributed to their urology group practice using an established algorithm that assesses annual billing patterns. ^12^

### 2.2 Health care delivery system determinants

**FIGURE 1:**
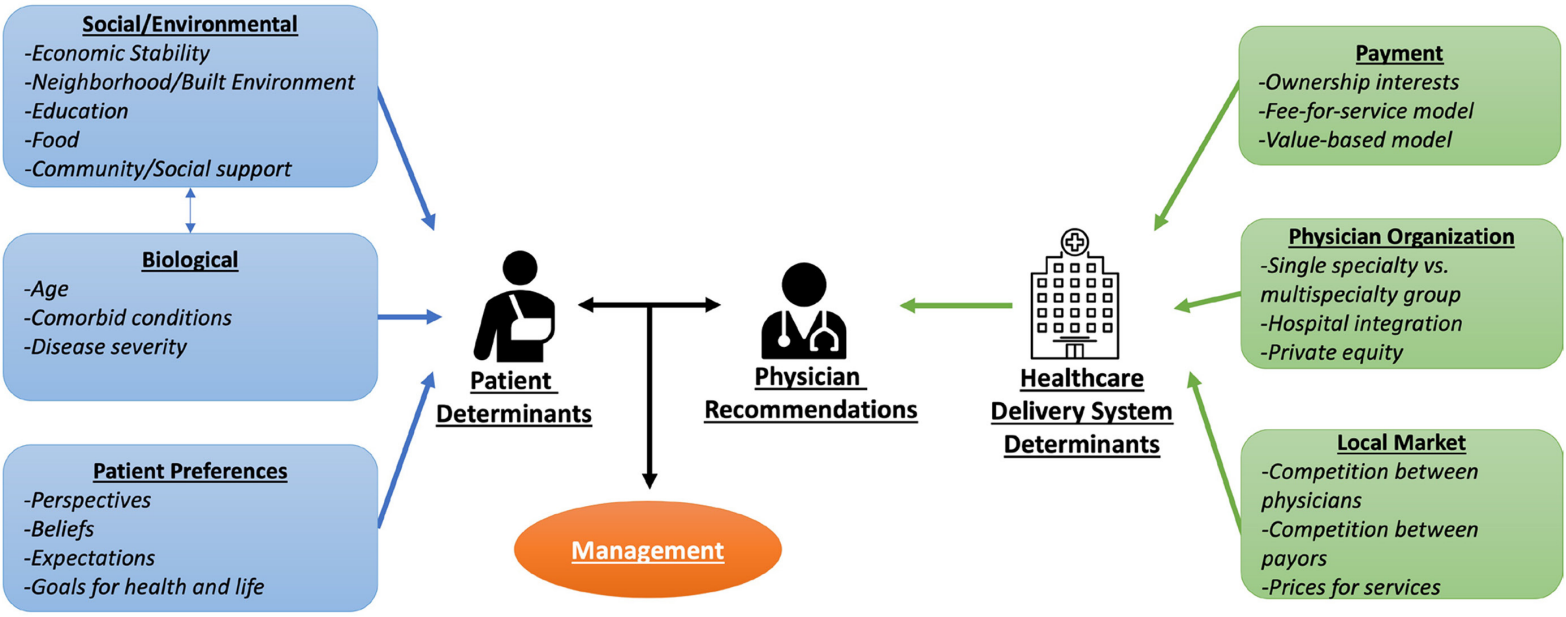
Conceptual model illustrating examples of patient and health care system determinants that may influence management of prostate cancer.

Based on our conceptual framework (Figure 1), there are numerous factors that may influence treatment—some are attributable to the patient and others to the broader health care delivery system. Differences in disease biology and patient physiology among men with newly diagnosed prostate cancer necessarily should inform the need for treatment. Some of these differences are measurable, reflect disease biology and patient physiology, and inform current clinical guidelines. On the contrary, health care delivery system determinants should play little role in deciding who gets treatment or how those opting for treatment get treated. To understand the contribution of health care delivery system determinants to variation in use of treatment, three practice‐level measures encompassing financial incentives, physician organization, and the health care market were developed empirically. First, urology practice ownership of radiation vaults was enumerated, as prior work demonstrated its strong association with use of treatment, due to associated payment incentives.^10^ Ownership was determined using an established and validated claims‐based algorithm that had 86.8% sensitivity and 93.6% specificity.^10^ Second, based on prior work demonstrating that treatment varies significantly with how urologists organize themselves,^10^ urology practices were characterized by their organization context as single specialty groups, multispecialty groups (i.e., groups including primary care physicians and in which urologists constituted fewer than 50% of physicians), or hospital affiliated groups (i.e., those employed by the hospital).^13^ Single specialty groups were further categorized based on size (i.e., small [1–2 urologists], medium [3–9 urologists], and large [10 or more urologists]).^14^ Third, practice‐level market competition was characterized due to its relationship with clinical demand and commercial prices, which are both associated with utilization.^13^ The competitiveness of the market in which a practice draws the majority of its patients was defined using the Herfindahl–Hirschman Index (HHI). This measure was calculated for each practice based on flow of patients to a given practice using a variable radius approach.^14^ Categories of practice‐level market competition (HHI <2,500), moderate (2,500 ≤ HHI ≤4,000), and low (HHI >4,000) based on thresholds set forth by the Federal Trade Commission and the Hospital Competition Act.^15^

### 2.3 Outcomes

The primary outcome of interest was treatment, measured at the patient level, and was inclusive of surgery, radiation, brachytherapy, or cryotherapy. Conservative management, defined as the absence of treatment, was inclusive of watchful waiting, active surveillance, and primary androgen deprivation. Men receiving androgen deprivation in the neoadjuvant or adjuvant setting (i.e., 6‐month period before or after treatment) were classified as having treatment.

We hypothesized that variation would be most pronounced when the benefits of treatment are the least clear, such as in men with the highest risk of noncancer mortality within 10 years of diagnosis. Based on clinical trials, guidelines have generally endorsed local treatment for men with a life expectancy of more than 10 years, as those with less were more likely to die from health conditions other than prostate cancer.^16^, ^17^ Therefore, the probability of death within 10‐years from noncancer causes was estimated using established methods and categorized as low (<25%), intermediate (25 to <50%), high (50 to <75%), and very high (≥75%).^10^

### 2.4 Statistical analysis

First, we compared patient characteristics across noncancer mortality risk groups using chi‐squared test for categorical variables and simple linear regression for continuous variables. Then we examined treatment trends as a function of noncancer mortality risk using the Cochrane–Armitage test.

Next, to visualize the degree of variation in use of treatment by noncancer mortality risk groups, we generated boxplots of estimated practice‐level use of treatment. To generate practice‐level use of treatment, we fit two‐level hierarchical logistic regression models (patients nested within practices) for each noncancer mortality risk group, with use of treatment as the primary outcome. The model was adjusted for patient‐level fixed effects including age, race, comorbidity using Klabunde methodology,^18^ socioeconomic status determined at the zip code level,^19^ and rural residence. We then generated average treatment probabilities for each practice by estimating the random effect for each practice using empirical Bayes predictions.^20^ This technique allows for shrinkage of the estimated rates of treatment to the overall mean rate based on the reliability of the estimate for each practice.^20^ For example, treatment rates for practices with higher case volume are more reliable and shrunken less towards the average treatment rate. Conversely, practices with small case volume are less reliable and shrunk more towards the average treatment probability.

Next, we sought to understand the relative contributions of patient and practice characteristics to the variation in use of treatment between practices. For each noncancer mortality risk group, we fit three hierarchical logistic regression models with a primary outcome of use of treatment and obtained the practice‐level variance for each model. Model 1 included only a random intercept for each practice and provided the baseline practice‐level variance. Model 2 included patient‐level covariates in addition to a practice‐level random intercept. Model 3 contained patient‐level covariates, practice‐level covariates, and a practice‐level random intercept. To determine the percent of practice‐level variance explained by patient characteristics, we calculated the proportional change in variance between Model 1 and Model 2 as:

$$\begin{array}{c} \text{Proportional Change in Variance}=\\ \frac{{\text{Variance}}_{\text{Model}\;1}-{\text{Variance}}_{\text{Model}\;2}}{{\text{Variance}}_{\text{Model}\;1}} \end{array}$$

To determine the percent of practice‐level variance explained by both patient and practice characteristics, we calculated the proportional change in variance between Model 1 and Model 3 using a similar equation as above.

As a means of further quantifying practice‐level variance, median odds ratios and confidence intervals were calculated using methodology described by Merlo et al.^21^ Median odds ratios represent the median increase in odds of treatment when a given patient is moved from a practice with a lower odds of treatment to a practice with higher odds of treatment, holding all other variables constant. In the context of this study, the median odds ratio would show the extent to which the probability of undergoing treatment is determined by the practice. The higher the median odds ratio, the more important the practice‐level effects are in determining differences in use of treatment.

All analyses were performed using Stata 17 (College Station, Tx) and SAS 9.4 (Cary, NC) statistical software. All tests were two‐sided with probability of type 1 error set at 0.05. This study was deemed not regulated by the Institutional Review Board.

## 3 RESULTS

We identified 62,507 men with newly diagnosed prostate cancer between 2014 and 2019. Patient characteristics differed by noncancer mortality risk (Table 1). Men with very high noncancer mortality risk were older (68 vs. 81, *p* < 0.001 from *t*‐test), had lower socioeconomic status (*p* < 0.001 from chi‐squared test), and had more comorbid conditions (*p* < 0.001 from chi‐squared test) compared to those with low noncancer mortality risk. Practice characteristics also varied, with men with higher risk of noncancer mortality more likely to be managed by small single specialty or hospital affiliated practices (*p* < 0.001 from chi‐squared test) and in areas of low market competition (*p* < 0.001 from chi‐squared test).

**TABLE 1:** Cohort demographic and managing practice characteristics, stratified by risk of noncancer mortality within 10‐years.

	Low	Intermediate	High	Very high	*p* Value
*N*	22,321	20,508	12,433	6,998	
Median age (IQR)	68 (67, 70)	73 (71, 75)	77 (73, 80)	81 (77, 85)	
Comorbidity score					
0	19,258 (86.3%)	11,117 (54.2%)	3,537 (28.4%)	613 (8.8%)	<0.001
1	2,676 (12%)	5,453 (26.6%)	2,957 (23.8%)	931 (13.3%)	
2+	387 (1.7%)	3,938 (19.2%)	5,939 (47.8%)	5,454 (78.0%)	
Socioeconomic status					
Low	4,997 (22.4%)	6,571 (32%)	4,522 (36.4%)	2,833 (40.5%)	<0.001
Medium	7,542 (33.8%)	7,300 (35.6%)	4,511 (36.3%)	2,415 (34.5%)	
High	9,782 (43.8%)	6,637 (32.4%)	3,400 (27.3%)	1,750 (25%)	
Race					
White	18,329 (82.1%)	17,500 (85.3%)	10,403 (83.7%)	5,871 (83.9%)	<0.001
Black	1,473 (6.6%)	2,041 (10%)	1,540 (12.4%)	901 (12.9%)	
Other	2,519 (11.3%)	967 (4.7%)	490 (3.9%)	226 (3.2%)	
Rural Residence	3,843 (17.2%)	4,302 (21%)	2,797 (22.5%)	1,464 (20.9%)	<0.001
Practice organization					
Small	2,605 (11.9%)	2,710 (13.5%)	1,942 (16%)	1,182 (17.4%)	<0.001
Medium	4,545 (20.7%)	4,454 (22.1%)	2,674 (22%)	1,468 (21.6%)	
Large	5,700 (26%)	5,086 (25.3%)	2,992 (24.7%)	1,708 (25.1%)	
MSG	7,389 (33.6%)	6,543 (32.5%)	3,851 (31.7%)	2,063 (30.4%)	
Hospital	17,25 (7.9%)	1,330 (6.6%)	676 (5.6%)	371 (5.5%)	
IMRT Owning Practice	5,467 (24.5%)	4,883 (23.8%)	2,985 (24%)	1,721 (24.6%)	0.320
Practice market competition					
High	1871 (8.5%)	1465 (7.3%)	817 (6.7%)	455 (6.7%)	<0.001
Moderate	6,274 (28.6%)	5,297 (26.4%)	3,087 (25.5%)	1,734 (25.6%)	
Low	13,782 (62.9%)	13,332 (66.3%)	8,214 (67.8%)	4,592 (67.7%)	

Abbreviations: IMRT, intensity modulated radiation therapy; MSG, multispecialty group.

**FIGURE 2:**
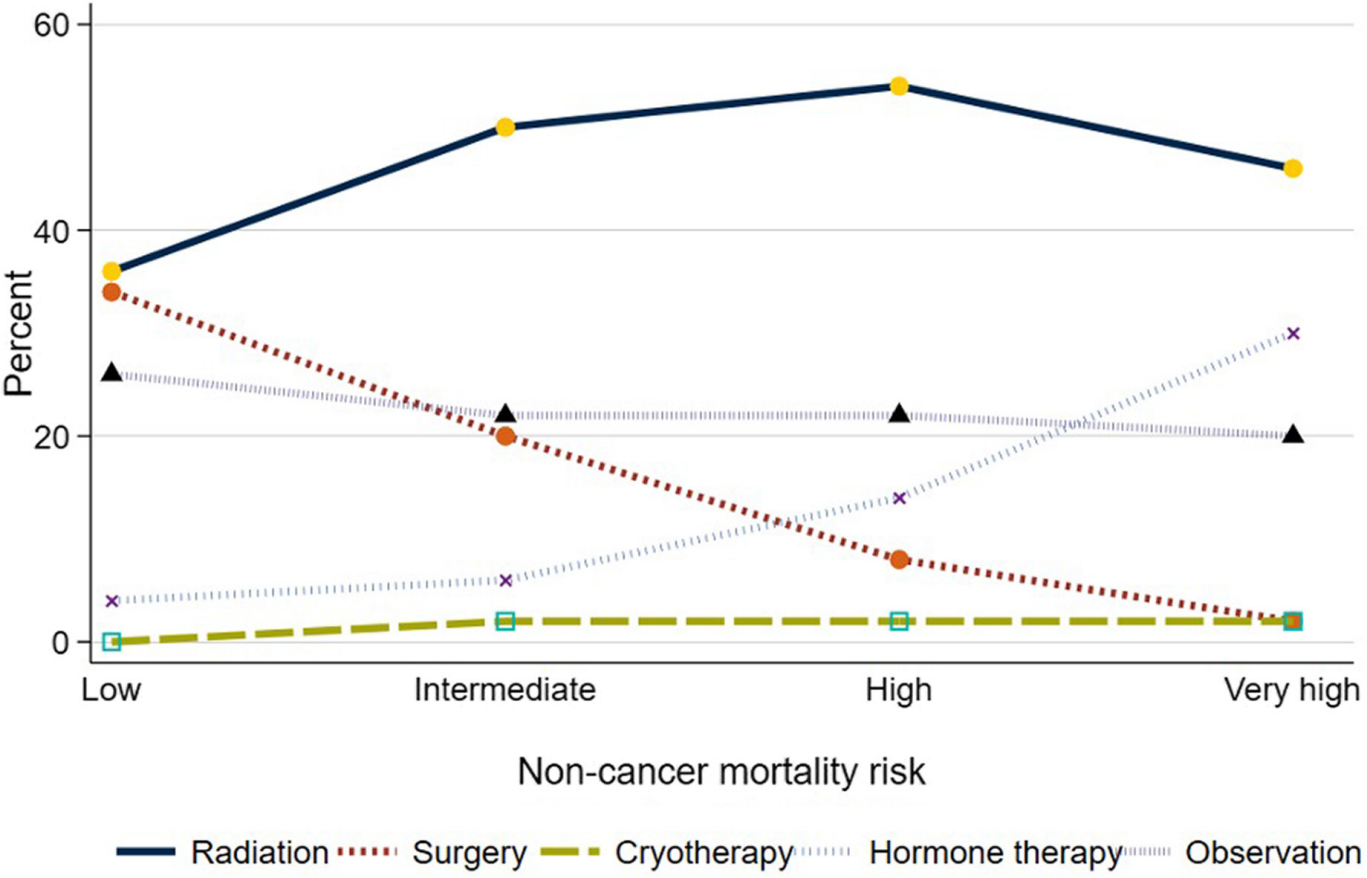
Initial management of newly diagnosed prostate cancer, stratified by risk of noncancer mortality within 10 years.

Management differed by noncancer mortality risk (Figure 2). Use of any local treatment declined from 71% to 51% (*p* < 0.001 for trend) as the risk of noncancer mortality increased from low to very high. Specifically, use of surgery declined from 34% to 3% (*p* < 0.001 for trend) while use of radiation increased from 37% to 46% (*p* < 0.001, for trend) as noncancer mortality risk increased.

**FIGURE 3:**
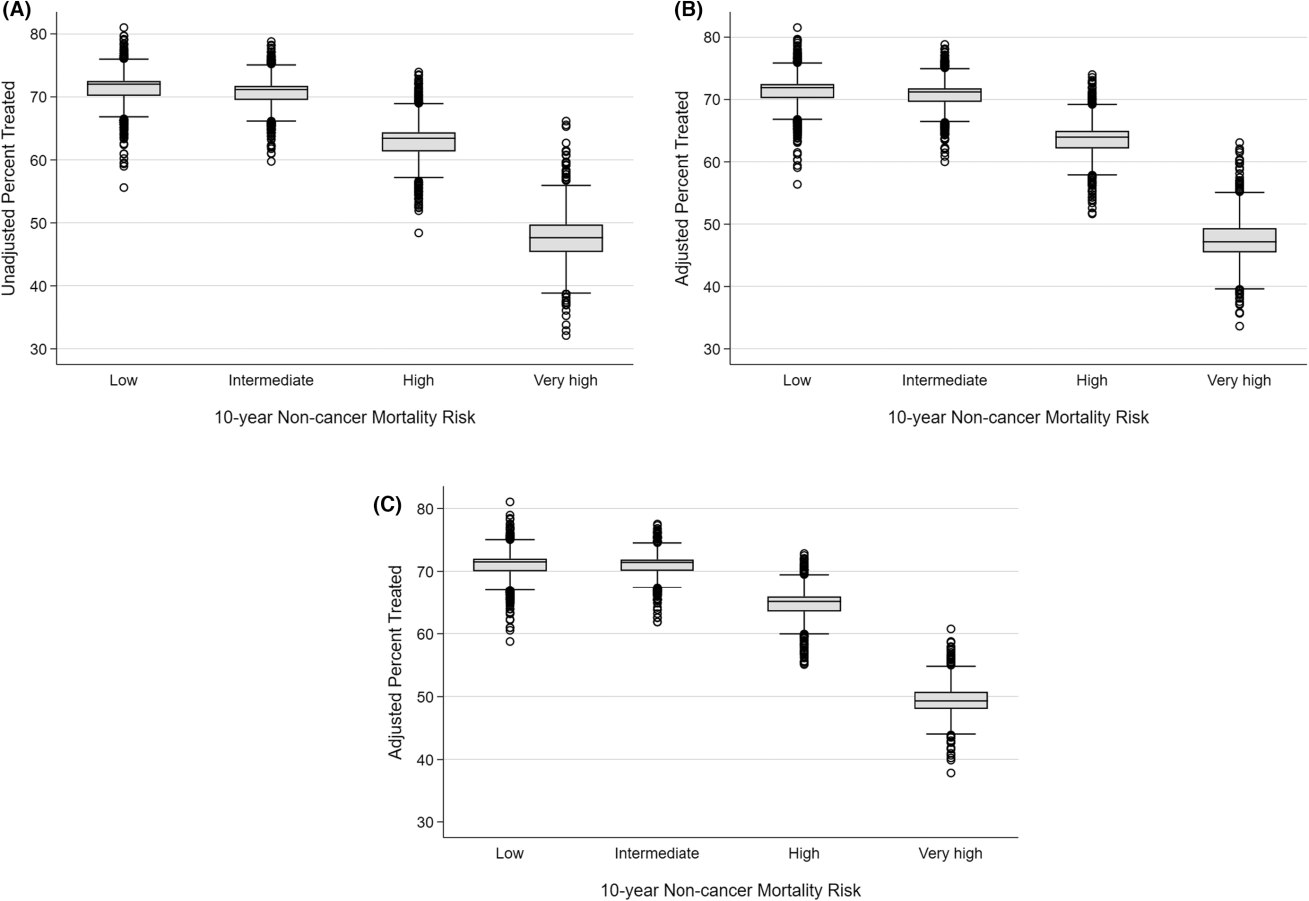
Practice‐level use of treatment, (A) adjusted for reliability only (i.e., case volume), (B) adjusted for reliability and patient factors stratified, and (C) adjusted for reliability, patient factors, and health care delivery system determinants, by risk of noncancer mortality within 10 years.

Hierarchical logistic regression models with a random intercept for practices were used to calculate practice‐level reliability adjusted use of treatment for each noncancer mortality risk group. The extent of variation between practices in use of treatment differed by risk of noncancer mortality (Figure 3). As shown in Supplemental Table S1, for men with low and intermediate noncancer mortality risk, the difference between the 1st and 99th percentile practices was 11% (low: 65% vs. 76%, intermediate: 65% vs. 76%). The differences in the probability of treatment in men with high and very high of noncancer mortality risk between practices in the 1st and 99th percentile was 15% and 20%, respectively (high risk: 56% vs. 71%, very high risk 39% vs. 59%). Adjustment for patient characteristics minimally reduced the variation across noncancer mortality risk groups (Figure 3B). Conversely, addition of health care delivery system determinants reduced the variation among the high and very high noncancer mortality risk groups (Figure 3C).

Next, the degree to which practice‐level variance was explained by patient and practice characteristics was measured across strata of noncancer mortality risk. As shown in Table 2, practice‐level variance increased with noncancer mortality risk, from 0.093 in men with low risk to 0.183 in men with very high risk. Addition of patient‐level factors explained a small share of the variance (0% in the low, 0% in the intermediate, 7.0% in the high, and 7.6% in the very high‐risk group). Addition of practice‐level factors explained 12.9% and 14.8% of the practice‐level variance among men with low and intermediate noncancer mortality risk, respectively. In men with high and very high noncancer mortality risk of addition of practice‐level factors explained 25.6% and 40.9%, respectively, of the variance in treatment.

**TABLE 2:** Measures of variance across models stratified by 10‐year noncancer mortality risk. Measures include variance, the proportional change in variance relative to model 1 (empty model), and the median odds ratio.

	Low	Intermediate	High	Very high
No of patients	22,321	20,508	12,433	6998
No of practices	2147	2239	2004	1614
Model 1: Empty				
Practice‐level variance	0.093	0.081	0.129	0.198
Proportional change in variance^a^	–	–	–	–
Median odds ratio (CI)^b^	1.34 (1.32, 1.35)	1.31 (1.30, 1.33)	1.41 (1.39, 1.42)	1.53 (1.50, 1.56)
Model 2: Patient variables				
Practice‐level variance	0.093	0.081	0.120	0.183
Proportional change in variance	0%	0%	−7.0%	−7.6%
Median odds ratio (CI)	1.34 (1.32, 1.35)	1.31 (1.30, 1.32)	1.39 (1.38, 1.40)	1.50 (1.48, 1.52)
Model 3: Patient and practice variables				
Practice variance	0.081	0.069	0.096	0.117
Proportional change in variance	−12.9%	−14.8%	−25.6	−40.9%
Median odds ratio (CI)	1.31 (1.30, 1.35)	1.29 (1.27, 1.29)	1.35 (1.33, 1.35)	1.39 (1.37, 1.40)

*Note*: Model 1: Model with random intercept for practice only. Model 2 Random intercept for practice and patient covariates: adjusted for age, year of dx, comorbidity score, race, socioeconomic class, Medicare dual eligibility, and rurality of residence. Model 3: Random intercept for practice, patient covariates, and practice covariates adjusts for above patient‐level variables and practice‐level variables: practice organization, radiation vault ownership, and market competition.

^a^ Proportional change in variance is relative to Model 1.

^b^ The median odds ratio quantifies the extent to which the probability of undergoing treatment is determined by the practice. A higher median odds ratio indicates that odds of treatment differ substantially across practices, while a lower median odds ratio indicates that the odds of treatment is relatively consistent across practices. The confidence intervals are obtained from parametric bootstrap with repeated draws from the estimated random intercept posterior distribution.

To further quantify variation in treatment, median odds ratios were calculated for each model. The median odds ratio was highest among men with very high risk of noncancer mortality and lowest among men with low risk of noncancer mortality (very high: median odds ratio: 1.53, 95% confidence interval [95% CI] 1.51–1.56 vs. low: median odds ratio: 1.34, 95% CI 1.32–1.35). In other words, if a man with a very high risk of noncancer mortality moved from a practice with low treatment rates to a practice with high treatment rates, his odds of receiving treatment would increase by 53%. Among men with low and intermediate risk of noncancer mortality, the median odds ratio changed minimally with addition of patient and practice factors. Conversely, among men with high and very high‐risk of noncancer mortality, the median odds ratio declined primarily after addition of practice‐level factors from 1.41 (95% CI 1.39–1.42) to 1.35 (95%CI 1.33–1.35) for high risk and 1.53 (95%CI 1.51–1.56) to 1.39 (95%CI 1.37–1.40) for very high risk.

## 4 DISCUSSION

In this study, we found variation in use of treatment increased with risk of noncancer mortality. At the lower end of the noncancer mortality risk spectrum, practice patterns are less variable, suggesting better consensus on how to manage these men. Conversely, at the higher end of the noncancer mortality risk spectrum, practice patterns are more variable, reflecting higher levels of medical uncertainty about management. Notably, characteristics of the delivery system, measured at the level of the urology practice, explained a larger share of the variation in treatment, particularly in settings in which the medical uncertainty of its value is greatest (i.e., in men with high and very high risk of noncancer mortality).

Variation in use of treatment between practices increased with risk of noncancer mortality. Practice patterns were less variable for men with low and intermediate risk of noncancer mortality, suggesting there may be less uncertainty regarding how these men should be managed. For young, healthy men, the decision to treat prostate cancer is likely to be driven by biological factors, such as tumor grade and stage.^22^, ^23^ These men tend to have the greatest improvement in overall survival following local treatment, in part due to the long time horizon over which benefits are realized.^21^, ^24^ The largest variation in use of treatment was among men with the highest risk of noncancer mortality. For older, sicker men, the benefits of treatment must be weighed against competing risks, which may result in a degree of clinical uncertainty regarding how these patients should be managed.^25^, ^26^ The variation in clinical guidelines may be reflective of this uncertainty. For instance, randomized trial data demonstrated survival benefits of treatment are realized after 10 years,^27^ leading clinical organizations to recommend against local treatment for men with less than 10 years of life expectancy—a concept that has been indoctrinated within urologic teaching.^28^ However, this paradigm has been increasingly questioned, with observational data demonstrating some benefit of treatment among older men with high‐grade disease.^29^, ^30^ The National Comprehensive Cancer Network, for example, has altered their recommendations, advocating for treatment of men with at least 5 years of life expectancy and high‐grade disease.^5^ Interestingly, this does not appear to have been universally adopted by all relevant organizations.^2^, ^31^ Additionally, variation in the treatment of these men may be further amplified by physicians' poor estimation of life expectancy.^6^, ^32^ Moreover, life expectancy estimates are both imprecise and inconsistent between and within physicians.^33^

Not only were practice patterns most variable among men with high and very high risk of noncancer mortality—for whom there is greater clinical uncertainty about the value of treatment—but differences in health care delivery system determinants measured at the level of the practice explained a large share of the variation. This phenomenon is consistent with early work examining geographic differences in use of surgery for numerous conditions, demonstrating that variation tended to be greatest in circumstances of clinical uncertainty.^9^ Importantly, these findings suggest features of the delivery system relating to financial incentives, physician organization, and the health care market are associated with treatment. The impact of these health care delivery system determinants is concerning because they may result in unwarranted treatment and morbidity for men who may not benefit. The precise mechanism by which these factors influence management of men with prostate cancer is not well understood. Prior work suggests that these factors may exert their influence through financial incentives, particularly in contexts in which treatment could be considered discretionary.^10^, ^11^ For instance, use of androgen deprivation therapy (which comprised up to 40% of urology practice revenue) in the 1990s rose eight fold among older men with low risk tumors—a group of men least likely to benefit from this therapy.^34^ Following cuts in payment for androgen deprivation therapy, the sharpest decline in its use occurred among these men.^35^

These findings must be considered in the context of several limitations. First, this data does not contain information on disease grade or stage, which certainly influence treatment decisions. However, it is unlikely that severity of disease would be imbalanced across large groups, such as urology practices, to meaningfully account for the variation observed. Furthermore, while we do not have information on disease severity, one of the main benefits of this data is that it is a nationally representative random sample. While other registries, such as SEER‐Medicare, may contain information on tumor grade and stage, prior work has shown that these data miss a significant number of practices and patients in Medicare, making it less generalizable and not truly nationally representative.^10^ Second, we estimated noncancer mortality from a model which may not account for all factors that influence mortality risk. However, the model has demonstrated high discrimination (*C*‐statistic = 0.82) and incorporates the patient factors that are known to be most strongly associated with mortality risk.^6^ Finally, we include several practice level characteristics that may influence treatment based on data from prior work,^10^, ^11^, ^13^ however, there may be other factors that also impact treatment decisions and thereby contribute to variation between practices. Regardless, our goal was to understand if health care delivery system determinants contribute to the observed variation in use of treatment, particularly in circumstances in which it would be considered discretionary. The characteristics we measured at the practice level encompassed domains plausibly important for health care delivery, including financial incentives, physician organization, and the health care market. Even with these focused measures, we demonstrated that they do account for a large share of the variation in use of treatment between practices among men with high and very high‐risk of noncancer mortality.

These limitations notwithstanding, our findings have important implications for patients and policymakers. The decision of whether to proceed with treatment or not can be difficult. While it should be based on biologic factors and shared decision making, the physician's influence is known to strongly impact treatment decisions.^7^ In circumstances of clinical uncertainty or when the decision to treat is discretionary (i.e., men with high or very high‐risk of noncancer mortality), these health care delivery system determinants can exert unwarranted influence and lead to unnecessary treatment. Importantly, treatment in these circumstances may exacerbate financial toxicity and lead to decisional regret, with potentially little added clinical benefit.^36^ Therefore, the variation attributable to practice‐level factors implies that interventions at the level of the practice may be effective in improving the quality of prostate cancer care. The previously cited example of androgen deprivation therapy is illustrative of this phenomenon in which policy that reduced payment for this treatment resulted in a significant decline in its use, only for circumstances in which there were no proven clinical benefits.^35^ Similarly, policy may act on these various health care delivery system determinants to improve the quality of care. For instance, while the increasing consolidation of physicians and hospitals is concerning, primarily due to the impact on prices and patient access,^37^ increased transparency of health care markets (i.e., through reporting of practice quality and prices) may help patients make more informed decisions of where to seek care. Additionally, policy may strengthen antitrust enforcement to prevent dominant practices or health systems from leveraging their market power to negotiate higher prices or limit patient choices.^38^ Similarly, promoting competition and directly incentivizing high value care through value‐based payment models that more precisely align incentives with high quality care can help ensure that changes to the delivery system do not lead to decreased quality.

## 5 CONCLUSION

Variation in use of treatment between urology practices increased with noncancer mortality risk. Health care delivery system determinants, measured at the level of the urology practice, explained a large share of the variation in treatment. This was most evident in clinical contexts with the highest amount of medical uncertainty (i.e., in men with high and very high‐risk of noncancer mortality). about the benefit of treatment.

## AUTHOR CONTRIBUTIONS

**Noah Krampe:** Conceptualization (equal); investigation (equal); methodology (equal); writing – original draft (equal). **Samuel R. Kaufman:** Data curation (equal); methodology (equal); writing – review and editing (equal). **Mary K. Oerline:** Data curation (equal); methodology (equal). **Dawson Hill:** Conceptualization (equal); writing – review and editing (equal). **Megan E.V. Caram:** Conceptualization (equal); supervision (equal); writing – review and editing (equal). **Vahakn B Shahinian:** Conceptualization (equal); funding acquisition (equal); investigation (equal); resources (equal); supervision (equal); writing – review and editing (equal). **Brent K Hollenbeck:** Conceptualization (equal); funding acquisition (equal); methodology (equal); supervision (equal); writing – review and editing (equal). **Avinash Maganty:** Conceptualization (equal); formal analysis (equal); methodology (equal); visualization (equal); writing – original draft (equal); writing – review and editing (equal).

## FUNDING INFORMATION

Avinash Maganty is supported by funding from the National Cancer Institute Postdoctoral Fellow Award F32 Grant F32 CA275021‐01. This work is supported by funding from a National Cancer Institute Grant R01CA269367–01 and by a Research Scholar Grant from the American Cancer Society (RSGI‐21‐097‐01‐HOPS).

## CONFLICT OF INTEREST STATEMENT

All authors have no disclosures or conflicts of interest.

## Supporting information


Table S1

## Data Availability

This study used Medicare claims data, provided by the Centers for Medicare & Medicaid Services (CMS) under license/by permission. Data may be shared on request to the corresponding author with permission of the CMS.
