# Supplementary material for: Health care delivery system contributions to management of newly diagnosed prostate cancer
Source: Cancer Med. 2023 Jul 20;12(16):17346–55. doi: 10.1002/cam4.6349 (PMC10501260; doi:10.1002/cam4.6349)
Supplement: Supplementary file 1 — Table S1 [file CAM4-12-17346-s001.docx]

**Supplemental Table 1.** Estimated percentiles of the distribution of practice rates of treatment of men of newly diagnosed prostate cancer, stratified by risk of non-cancer mortality within 10 years. Unadjusted rates were derived from a multilevel model with a practice level random intercept without inclusion of covariates. Adjusted rates were determined by addition of patient-level and practice-level covariates to the empty model.

|  | Percentile | | | | | | |
| --- | --- | --- | --- | --- | --- | --- | --- |
|  | **1^st^** | **10^th^** | **25^th^** | **50^th^** | **75^th^** | **90^th^** | **99^th^** |
| Low |  |  |  |  |  |  |  |
| *Unadjusted* | 64.6 | 68.3 | 69.6 | 71.4 | 72.0 | 73.1 | 76.0 |
| *Adjusted for patient covariates* | 64.6 | 68.3 | 69.6 | 71.4 | 72.0 | 73.0 | 76.0 |
| *Adjusted for patient and practice covariates* | 65.5 | 68.9 | 70.0 | 71.5 | 72.0 | 72.9 | 75.6 |
| Intermediate |  |  |  |  |  |  |  |
| *Unadjusted* | 65.4 | 68.7 | 69.8 | 71.4 | 71.9 | 72.8 | 75.6 |
| *Adjusted for patient and practice covariates* | 65.5 | 68.7 | 69.9 | 71.4 | 72.0 | 72.8 | 75.6 |
| *Adjusted for patient and practice covariates* | 66.1 | 69.1 | 70.1 | 71.4 | 71.8 | 73.7 | 75.2 |
| High |  |  |  |  |  |  |  |
| *Unadjusted* | 56.2 | 60.2 | 62.0 | 64.1 | 65.0 | 66.8 | 71.0 |
| *Adjusted for patient and practice covariates* | 57.3 | 61.1 | 62.6 | 64.6 | 65.6 | 67.1 | 70.9 |
| *Adjusted for patient and practice covariates* | 57.5 | 61.2 | 62.7 | 64.6 | 67.1 | 68.3 | 70.9 |
| Very high |  |  |  |  |  |  |  |
| *Unadjusted* | 39.0 | 44.1 | 46.2 | 48.6 | 50.9 | 53.1 | 59.2 |
| *Adjusted for patient covariates* | 40.0 | 44.6 | 46.3 | 48.3 | 50.6 | 52.5 | 58.4 |
| *Adjusted for patient and practice covariates* | 43.8 | 47.0 | 48.1 | 49.3 | 50.7 | 51.8 | 55.5 |
